# Association of *p*-phenylenediamine exposure with alterations of pulmonary function, pruritus and health-related quality of life in hair dye factory workers: a cross-sectional study

**DOI:** 10.1038/s41598-023-29721-7

**Published:** 2023-02-14

**Authors:** Ming Zhang, Meibian Zhang, Qiang Zeng, Dafeng Lin, Naixing Zhang

**Affiliations:** 1 Shenzhen Prevention and Treatment Center for Occupational Diseases, Shenzhen, 518020 People’s Republic of China; 2 Tianjin Centers for Disease Control and Prevention, Tianjin, 300011 People’s Republic of China; 3 National Institute of Occupational Health and Poison Control, Chinese Center for Disease Control and Prevention, Beijing, 100050 People’s Republic of China

**Keywords:** Environmental sciences, Natural hazards, Risk factors

## Abstract

*P*-Phenylenediamine (PPD) is a common component of hair dye, which can cause skin contact allergy and asthma with impaired pulmonary function. However, the adverse effects of occupational exposure to different dose PPD was rarely mentioned. We recruited 124 workers from a hair dye factory to explore the association of occupational PPD exposure on pulmonary function, pruritus and health related quality of life (HRQL). We categorized exposure to PPD into 3 levels: lower exposure group (< 0.00001 mg/m^3^); middle exposure group (0.00001–0.00033 mg/m^3^); higher exposure group (0.00033–0.047 mg/m^3^). The HRQL and subjective pruritus of the workers were assessed by the short form 36 health survey (SF-36) and Visual analogue scale (VAS) of pruritus, respectively. In the high PPD-exposed group, the percentage of FEV1 (FEV1%) was lower in higher exposure group compared with lower exposure group. The FEV1/FVC was also lower in comparison to the higher exposure and middle exposure groups (*p* < 0.05). PPD levels were negatively correlated with vitality and mental health (*p* < 0.01). The structural equation model showed the positive effects of PPD on VAS level (β = 0.213, *p* < 0.001), and indicated partly negtive effects of PPD on total score of SF-36 (β = − 0.465, *p* = 0.002), respectively. Our results indicate that occupational exposure to PPD might be associated with pulmonary function impairment, poor HRQL, and subjective pruritus of the workers.

## Introduction

*P*-Phenylenediamine (PPD), a derivative of *p*-nitroanaline, is found in the plastic and chemical industries as byproducts of manufacturing. PPD has a strong protein binding ability to penetrate deeply into the hair. It is widely used as an oxidizable hair dye for its free availability and low cost. In addition, this chemical is used as a substitute for henna and in the manufacturing of rubber and certain polymers^1^. PPD is highly toxic with local and systemic toxic effects when taken by mouth, and the outcome depends mainly on the dose taken. Many studies have shown that PPD has multiple organ system toxicity, which can cause skin allergies and itching, cardiotoxicity, and urinary toxicity. Among these health hazards, the most common is the skin toxicity and respiratory damage in low-dose exposure of PPD. In addition, PPD used in hair dyes has been considered as an important cause of hair dye allergy, which can lead to skin allergy including severe contact dermatitis^2,3^.

In 1939, PPD was first defined as a strong allergen and included in the standard antigen group of the North American and European epidermal patch tests. According to European Cosmetic Directive (76/768/EEC), PPD’s maximum permissible concentration in hair coloring is 2%^4^. It is reported that PPD causes erythema, scaling, itching or edema in less than 1% individuals of the general population after dying the hair^5^. Occupational exposure to PPD foam, during its production or application in manufacturing of various products, may result in health problems affecting airway and skin. Both allergic contact dermatitis (ACD) and irritant contact dermatitis (ICD) have been reported^6,7^. The incidence of PPD related ACD, an occupational-related, positive patch test reaction to PPD, is about 4.80% in outpatients with occupational exposure to PPD such as hairdressers, barbers^8^. PPD may also cause damage to the lungs. Induction routines and challenge patches with 0.001% to 10% PPD sensitized 56% to 100% of guinea pigs on test. Massive peribronchial infiltrates of eosinophils were observed in guinea pigs after intrapulmonary injection of an aqueous solution containing 1% PPD^9^. The serious level of PPD allergy is mostly determined by the exposure concentration and duration^10^. It is reported that PPD causes erythema, scaling, itching or edema in less than 1% individuals of the general population after dying the hair^5^. Moreover, long-term occupational exposure to PPD may lead to chronic renal impairment in workers^11^. Oxidized products of PPD might also have potential carcinogenicity to humans. Association has been observed between hair dye use and non-Hodgkin’s lymphoma, multiple myeloma, acute leukemia and bladder cancer^12–14^.

As aforementioned, occupational exposure to PPD can result in health problems such as skin allergies and pulmonary function changes^6^. However, previous studies have mostly conducted research on ordinary people or emergency patients, and the studies of occupational groups only focused on individual case reports of hairdressers. So far, no research has been conducted on workers who are working with exposure PPD. And examination including peripheral blood, pulmonary function has been presented as an easily-used method reflecting the physical condition^15,16^. In addition, there are few studies evaluating the effect of the PPD exposure on the itching of the skin of the workers. In order to evaluate skin discomfort and correlate it with the effects on the individual, there is also a need for methods to determine the clinical severity of people. Compared with patch-test to PPD allergy in previous research, the Visual analogue scale (VAS) could evaluate the skin discomfort without an obvious allergic reaction^8,17^. Whether these adverse effects (pulmonary dysfunction and pruritus) of PPD lead to a decline in their life quality, and then interfere with their activities of daily living and work is noteworthy. Health-related quality of life (HRQL) is a broad and multidimensional measure that assesses an individual’s perception of physiological function, mental ability and social adaptability. HRQL can be influenced by health status, medical interventions, personal beliefs, social environments, economic circumstances and cultural backgrounds^18^. The purpose of this study aimed to investigate the exposure status and evaluate pulmonary function of low dose PPD-exposed workers in hair dye production, and evaluate subjective pruritus and HRQL by the short form 36 health survey (SF-36) and Visual analogue scale (VAS) of pruritus, respectively. We believe that this study will provide useful information for health care policy makers and health care providers so as to develop better strategies on health education programs for PPD-exposed control.

## Methods

### Study population

The cross-sectional study was based on data from a hair dye factory containing PPD in Zhejiang Province of China. The occupational health management in this company is standardized, possessing protective measures that can prevent contact with dangerous substance includes masks, protective clothing, boots and overshoes. The factory employees were first coded and then randomly sampled by computer generated random numbers. The employees are engaged in configuring raw material (*n* = 9), adding chemical (*n* = 9), adding material into the jar (*n* = 28), packaging and transporting (*n* = 50), laboratory staffs (*n* = 4), accounting (*n* = 6), cleaning and logistics (*n* = 13) and managing (*n* = 5). The sample had 68 men and 56 women. No participants had history of allergic reaction. They were asked to fill out a demographic questionnaire which included the age, gender, area of residence, telephone number, working age, educational condition, smoking status and hair dye use status. All participants have signed informed consent, and ethical approval was granted by the Ethics Committee of Tianjin Center for Disease Control and Prevention.

### Measurement of PPD concentration

Referenced to the method 5013 of the U.S. National Institute for Occupational and Safety (NOISH)^19^, the PPD in the air of various working environments and workers of different work types (simple random sampling of 3–5 individuals per type of work) were sampled by Gilair-3 air sampling pump (Gilian, SENSIDYNE, St. Petersburg, FL, USA), collected with glass fiber filters coated with dilute sulfuric acid (Nantong Jinnan Glass Instrument, Jiangsu, China), and extracted with an aqueous EDTA solution (Guangfu Fine Chemical Research Institute, Tianjin, China), and then determined by high performance liquid chromatography (HPLC, SHIMADZU, Inc., Kyoto, Japan) with the analytical column of Eclipse SB-C18 (150 mm × 4.6 mm × 5 μm) and the mobile phase of mixed solution of disodium hydrogen phosphate solution and acetonitrile with a flow rate of 2.0 mL/min. The detection wavelength was 240 nm and the injection volume was 5 μL.

### Self-reported measures of life condition

The subjects completed two self-reported assessment for life condition: SF-36 and VAS of pruritus.

The quality of life was assessed by SF-36, derived from the health survey results of the Medical Outcomes Study (MOS) in 1988. It has been translated into Chinese in 1991 and its reliability and validity has been verified in Chinese population after considering cultural adaptation, validation, and normalization^20^. SF-36 measured eight dimensions of health including physical functioning, role-physical, bodily pain, general health, vitality, social functioning, role-emotional and mental health. In addition to these dimensions, SF-36 also considered reported health transition scaled scores^21^. Each scale was directly transformed into a score from 0 to 100 after modifying the weighing coefficient in each question. The lower score meant the worse health status, whereas the higher the score the less possible disability^22^. The Cronbach's α values for each dimension of SF-36 were calculated to be greater than 0.7, indicating that the internal consistency of the scale is good in occupational workers. After factor analysis, the KMO value was 0.625 and Bartlett's test result show *p* value was less than 0.001, indicating that the structural validity was good. Therefore, this scale had suitable reliability and validity for the occupational population.

The extent of subjective pruritus was assessed by VAS of pruritus^23^. It has been widely used because of a simple structure and flexible operation. Participants could mark a score for a 100 mm horizontal line on the questionnaire to represent their subjective feelings of cutaneous itching. The benchmark descriptors for each level were as follows: 1 level: 0 mm to 10 mm (i.e., no itching symptoms), “no or almost no itch”; 2 level: 10 mm to 40 mm (i.e., slight itching symptoms), “occasional restless sensations that do not necessarily induce scratching behavior”; 3 level: 40 mm to 70 mm (i.e., moderate itching symptoms), “significant scratching and the itch disrupts sleep, and although the patient can go back into sleep after scratching”; 4 level: more than 70 mm (i.e., fierce itching symptoms), “unbearable itch causes scratching and scratching almost deprived of sleep, the patient keeps scratching ceaselessly”. Because of the subjectivity of pruritus, we regard all patients with pruritus symptoms more than 0 mm as having pruritus symptoms.

### Biochemical detection

The levels of alanine transarninase (ALT), aspartate aminotransferase (AST), total protein (TP), albumin (ALB), alkaline phosphatase (ALP), total bilirubin (TBI) in serum were detected using commercial test kits (Jiancheng Bioengineering Ltd., Nanjing, China).

### Hematologic detection

Red blood cell (RBC), white blood cell (WBC), hemoglobin (HGB) and platelet (PLT) were detected using commercial test kits (Jiancheng Bioengineering Ltd., Nanjing, China).

### Pulmonary function examination

The portable spirometer (Contec SP10, Contec Ltd., China) was used to measure the capacity and the volume of lungs..Measurement indexes included forced vital capacity (FVC), forced expiratory volume in one second (FEV1), and ratio of FEV1 to FVC (FEV1/FVC), etc. Abnormal pulmonary function was defined as: FVC% (percentage of forced vital capacity, FVC measured value/FVC predicted value) < 80%; FEV1% (percentage of forced expiratory volume in 1 s, FEV1 measured value/FEV1 predicted value) < 75%; FEV1/FVC%, FEV1 measured value/FVC measured value < 70%. The predictive values of FVC or FEV1 was automatically calculated by the pulmonary function analyzer according to age, gender, height, weight and race. A disposable mouthpiece was used for each participant. The participant inhaled maximally through the nose until the lungs were full. Afterward, the participant placed the spirometer through the disposable mouthpiece in his/her mouth, with lips sealed tightly around the mouthpiece while holding the lungs full^24^. The participant was instructed to exhale forcefully as long as possible into the spirometer until no air could be exhaled. A minimum of three trials was done and the values of FEV1, FVC and FEV1/FVC were obtained.

### Statistical analysis

Statistical analysis was conducted using SPSS package (Version 20.0) unless otherwise specified. Numerical variables were reported as mean ± SD. The Chi-square test and Chi-square after Yates continuity correction test were used to compare the differences of demographic situation and working age, educational, smoking and hair dye use between different groups. The covariance analysis and Bonferroni test were performed for comparing interesting variables in different groups after controlling for the confounding factors, and adjusted *P* < 0.017 (two-tailed) was considered statistically significant, taking the multiple comparisons into consideration. Covariates included gender, ethnic group, working age, education condition and recent hair dye history. Due to the co-linearity of working age and age, this study only adjusted working age as a covariate. The influencing factors of VAS levels in different groups were studied by logistic regression. Before the multivariate analysis, the Variance Inflation Factor was calculated to rule out the effects of multicollinearity. We performed correction test of collinearity and used logistic regression to estimate the odds ratio (OR) and its 95% confidence interval (95% CI) for the association of VAS level with hematologic indexes and some factor scores in SF-36. *p* < 0.05 (two-tailed) was considered statistically significant in the final models. Structural equation modeling (SEM), which can evaluate the relationship between variables, was performed with the AMOSE software. Cutoff criteria for good model fit were attained from extant literature; Satorra-Bentler χ^2^/df values < 3.0 were considered good; Goodness of Fit Index (GFI) values > 0.90 and Adjusted Goodness of Fit Index (AGFI) > 0.90 were considered reasonable; Root Mean Square Error of Approximation (RMSEA) values < 0.10, Incremental Fit Index (IFI) and Comparative Fit Index (CFI) values > 0.90, parsimony goodness-of-fit index (PGFI) > 0.50 were considered good. Consistent Akaike Information Criterion (CAIC)_default_ < CAIC_independence_ and CAIC_default_ < CAIC_saturated_ were considered good^25^. Missing data was replaced by means of the scores in the different groups.

### Approval of the research protocol

All procedures performed in the study involving human participants were in accordance with the ethical standards of the Ethics Committee of Tianjin Center for Disease Control and Prevention and with the 1975 Declaration of Helsinki and its later amendments or comparable ethical standards. The study was approved by the Ethics Committee of Tianjin Center for Disease Control and Prevention.

### Informed consent

Informed consent was obtained from all individual participants included in the study or their legal guardians.

## Results

### General characteristics of study population

The PPD exposure concentrations were cut into three levels (lower level: < 0.00001 mg/m^3^; middle level: 0.00001–0.00033 mg/m^3^; higher level: 0.00033–0.047 mg/m^3^). Subsequently, the 124 subjects were divided into three groups: lower dose PPD exposure group (24 workers), middle dose PPD exposure group (50 workers), and higher dose PPD exposure group (50 workers). The lower exposure group included financial staffs, support staffs, administrative personals and cleaners (< 0.00001 mg/m^3^). The packaging workers were designated as the middle exposure group (0.00001–0.00089 mg/m^3^), while the workers who were involved in raw material, adding chemical, adding material into the jar, and laboratory staffs were designated as the higher exposure group (0.00089–0.19 mg/m^3^) (Supplemental Table 1).

The distributions of demographic characteristics among the three groups were showed in Table 1. Higher exposure group, middle exposure group and lower exposure group statistically differed in gender, ethnic group, working age, education condition and hair dye history (*P* < 0.05). The male constituent ratio was higher in the higher exposure group (82%) than in the middle exposure group (36%) and the lower exposure group (37.5%). Among the three groups, the proportion of the Han nationality in middle exposure group (80%) was lower than the other groups. The number of workers with working age less than 5 years was the highest in the middle exposure group (54%). In the term of educational condition, the proportion of primary school and lower level was lower in the lower exposure group than the other two groups. The hair dye history in the lower exposure group (16.67%) had a lower constituent ratio than the higher exposure group (54%).

**Table 1:** General characteristics of the study subjects grouped by PPD exposure.

Variable	Lower exposure (n = 24)	Middle exposure (n = 50)	Higher exposure (n = 50)	Chi square/F	*p* value
PPD exposure concentration (mg/m^3^)	< 0.00001	0.00001–0.00033	0.00033–0.047		
Age (years)	41.29 ± 6.27	34.18 ± 9.00	39.86 ± 8.23	8.596^b^	< 0.001
Working age (years)	18.25 ± 10.66	7.04 ± 7.87	11.12 ± 7.49	14.727^b^	< 0.001
Gender					
Male	9 (38%)	18 (36%)	41 (82%)	24.973	< 0.001
Female	15 (62%)	32 (64%)	9 (18%)		
Ethnic group					
Han	1 (4%)	10 (20%)	3 (6%)		
Others	23 (96%)	40 (80%)	47 (94%)	5.578^a^	0.044
Marital status					
Married	18 (75%)	35 (70%)	40 (80%)	1.333	0.513
Others (unmarried and divorced)	6 (25%)	15 (30%)	10 (20%)		
Educational condition					
Junior high school and lower level	5 (21%)	35 (70%)	25 (50%)	15.914	< 0.001
Senior middle school level and higher	19 (79%)	15 (30%)	25 (50%)		
Cigarette smoking					
Yes	13 (54%)	25 (50%)	26 (52%)	0.118	0.943
No	11 (46%)	25 (50%)	24 (48%)		
Hair dye history					
Yes	4 (17%)	16 (32%)	27 (54%)	10.843	0.004
No	20 (83%)	34 (68%)	23 (46%)		
Body mass index (BMI) (kg/m^2^)					
< 25	15 (62%)	39 (78%)	28 (56%)	5.577	0.062
≥ 25	9 (38%)	11 (22%)	22 (44%)		

The most widely used way to estimate body fat is the body mass index (BMI)—body weight normalized by height squared (kg/m^2^). Being a very simple and inexpensive method, it is the basis for WHO’s definition of overweight (25 ≤ BMI < 30) and obesity (BMI ≥ 30).

^a^With Yates continuity correction.

^b^One-Way ANOVA.

### Changes of hematologic indexes of the study participants

The hematologic indexes results among the three groups were in the normal count ranges. After adjustments for gender, ethnic group, working age, education condition and recent hair dye history, the covariance analysis showed that in the higher exposure group, total white blood cell count was lower (*p* < 0.001), while basophils and eosinophils were higher (*p* < 0.05) than that in the lower and middle exposure groups. Besides, compared to the middle exposure group, workers in the higher exposure group presented a higher neutrophil and hemoglobin level. No differences were observed for other hematologic indexes indices among the three groups (Table 2).

**Table 2:** Covariance analysis results of serum biochemical indexes (mean ± SD).

Variable	Lower exposure (n = 24)	Middle exposure (n = 50)	Higher exposure (n = 50)	F	*p value*
White blood cell count (10^9^/L)	6.929 ± 1.243	6.656 ± 1.088^#^	5.206 ± 1.209*	8.569	< 0.001
Neutrophil (10^9^/L)	3.951 ± 0.914	3.423 ± 0.291*^#^	3.949 ± 0.911	2.803	0.010
Lymphocyte (10^9^/L)	1.839 ± 0.489	1.836 ± 0.480	1.925 ± 0.541	0.706	0.667
Basophils (10^9^/L)	0.015 ± 0.010	0.015 ± 0.014^#^	0.040 ± 0.015*	16.053	< 0.001
Monocytes (10^9^/L)	0.350 ± 0.078	0.342 ± 0.050	0.334 ± 0.063	0.718	0.657
Eosinophils (10^9^/L)	0.157 ± 0.112	0.212 ± 0.078*^#^	0.363 ± 0.102*	16.505	< 0.001
Red blood cell count (10^12^/L)	5.017 ± 0.392	5.122 ± 0.413	5.080 ± 0.420	0.379	0.913
Hemoglobin (g/L)	142.920 ± 13.247	140.780 ± 12.621^#^	148.160 ± 13.577	13.364	< 0.001
Mean corpuscular volume (fL)	85.670 ± 10.516	86.380 ± 10.463	85.560 ± 10.695	0.643	0.720
Mean corpuscular hemoglobin concentration (g/L)	340.880 ± 11.449	339.540 ± 8.779	338.400 ± 7.902	0.756	0.625
Mean platelet volume (fL)	9.370 ± 1.075	9.650 ± 0.497	9.480 ± 0.945	1.065	0.391
Platelet volume distribution width	11.996 ± 2.146	11.770 ± 1.668	11.796 ± 1.897	0.849	0.549
The average amount of Hb in red blood cells (pg)	28.920 ± 4.106	28.940 ± 4.043	29.720 ± 3.552	0.486	0.843
Platelet count (10^9^/L)	234.040 ± 45.108	237.940 ± 45.488	238.320 ± 43.507	1.105	0.365
Plateletcrit (PTC)	0.271 ± 0.042	0.258 ± 0.042	0.261 ± 0.045	0.639	0.723

The covariant variables including gender, ethnic group, working age, education condition and recent hair dye history.

*P < 0.05, comparison of the higher and middle exposure group with the lower exposure group by Bonferroni method.

^#^*P* < 0.05, comparison between the higher exposure group and middle exposure group by Bonferroni method.

### Alteration of pulmonary function of the study participants

After adjustments for gender, ethnic group, working age, education condition and recent hair dye history, the spirometry parameters of the three groups were significantly different. The pulmonary function indices like FEV1% and FEV1/FVC% of the higher exposure group were significantly lower than those of the lower exposure group (*p* < 0.05). In the higher exposure group, FVC% was higher, while FEV1/FVC% was lower than that in the middle exposure group (*p* < 0.05). In addition, compared to the lower exposure group, workers in the middle exposure group presented the lower FVC% and FEV1% (*p* < 0.05) (Table 3).

**Table 3:** Covariance analysis of FVC%, FEV1%, FEV1/FVC% in different PPD-exposure group (mean ± SD).

Variable	Lower exposure (n = 24)	Middle exposure (n = 50)	Higher exposure (n = 50)	F	*P value*
FVC%	103.044 ± 9.498	89.076 ± 11.806*^#^	101.399 ± 9.409	7.519	< 0.001
FEV1%	104.665 ± 12.289	91.772 ± 6.261*	92.483 ± 10.300*	8.791	< 0.001
FEV1/FVC%	102.215 ± 14.040	104.700 ± 14.779^#^	91.889 ± 12.737*	6.855	< 0.001

Measurement indexes of capacity and the volume of lung include forced vital capacity (FVC), forced expiratory volume in one second (FEV1), and ratio of FEV1 to FVC (FEV1/FVC), etc. Abnormal pulmonary function is defined as: FVC% (percentage of forced vital capacity, FVC measured value/FVC predicted value) < 80%; FEV1% (percentage of forced expiratory volume in 1 s, FEV1 measured value/FEV1 predicted value) < 75%; FEV1/FVC%, FEV1 measured value/FVC measured value < 70%. The predictive values of FVC or FEV1 is automatically calculated by the pulmonary function analyzer according to age, gender, height, weight and race.

*P < 0.05, comparison of the higher and middle exposure group with the lower exposure group by Bonferroni method.

^#^*P* < 0.05, comparison between the higher exposure group and middle exposure group by Bonferroni method.

The covariant variables including gender, ethnic group, working age, education condition and recent hair dye history.

### Self-reported of physical subjective feelings of the study participants

After excluding the influence of gender, ethnic group, working age, education condition and recent hair dye history, covariance analysis indicated that means for role-physical (*p* = 0.023), bodily pain (*p* < 0.001), general health (*p* < 0.001), vitality (*p* < 0.001), mental health (*p* < 0.001), health transition (*p* < 0.001) and total scores were all significantly different among the three groups (Table 4). The means for bodily pain, general health and health transition in the middle exposure group were lower than the lower exposure group and the higher exposure group (*p* < 0.05). Besides, the mean for mental health in the higher exposure group and role-physical in the middle exposure group was lowest of all groups (*p* < 0.05). Higher concentration PPD also resulted in a decreased vitality score compared to the lower exposure group. For the total score, the score for the lower exposure group was higher than the other groups, and the higher exposure group was higher than the middle exposure group (*p* < 0.05).

**Table 4:** Covariance analysis results of SF-36 (mean ± SD).

Variable	PF	RP	BP	GH	VT	SF	RE	MH	HT	Total score
Lower exposure (n = 24)	98.13 ± 2.88	96.09 ± 6.60	97.25 ± 8.77	92.0 ± 10.62	72.08 ± 9.55	92.71 ± 9.69	93.06 ± 9.08	61.5 ± 5.25	85.40 ± 24.36	788.32 ± 48.52
Middle exposure (n = 50)	97.40 ± 3.81	94.88 ± 9.25^#^	83.60 ± 21.01*^#^	78.4 ± 13.93*^#^	65.30 ± 14.16	91.20 ± 12.94	95.83 ± 8.29	59.04 ± 6.18^#^	59.50 ± 19.49*^#^	725.29 ± 47.11*^#^
Higher exposure (n = 50)	98.50 ± 2.31	96.87 ± 5.54	97.37 ± 5.23	88.20 ± 16.96	62.30 ± 11.91*	87.25 ± 15.04	94.3 ± 10.84	55.12 ± 8.68*	72.50 ± 22.73	752.45.32 ± 47.52*
F	1.276	2.444	8.972	6.983	5.51	1.632	0.617	4.477	6.163	3.780
*P*	0.268	0.023	< 0.001	< 0.001	< 0.001	0.133	0.741	< 0.001	< 0.001	0.026

The covariant variables including gender, ethnic group, working age, education condition and recent hair dye history.

SF-36: the short form 36 health survey; PF: physical functioning; RP: role-physical; BP: body pain; GH: general health; VT: vitality; SF: social functioning; RE: role-emotional; MH: mental health; HT: health transition.

*P < 0.05, comparison of the higher and middle exposure group with the lower exposure group by Bonferroni method.

^#^*P* < 0.05, comparison between the higher exposure group and middle exposure group by Bonferroni method.

The results of multi-variable logistic regression analysis for VAS levels with some demographic variables, SF-36 factors were showed in Table 5. It found that men were less likely to present high VAS levels than women (OR 0.114, 95%CI 0.027, 0.482, *p* = 0.003). The worker who works in a higher concentration PPD atmosphere significantly increased the probability of VAS level (OR 9.394, 95%CI 1.710, 51.622, *p* = 0.010). Individuals without a hair dye history had almost one tenth the probability of not (OR 0.091, 95% CI 0.021, 0.385, *p* = 0.001). And workers with senior middle school and higher education have lower level of VAS (OR 0.041, 0.033 respectively; both* p* < 0.05). As for SF-36 scores, workers with the higher BP score have higher VAS level (OR 1.056, 95% CI 1.008, 1.106, *p* = 0.021), and with higher GH score have lower VAS level (OR 0.950, 95% CI 0.904, 0.998, *p* = 0.040).

**Table 5:** Multiple Logistic regression analysis of VAS level with hematologic indexes and some factor scores in SF-36.

	β	${\overline{\text{S}}}_{{\text{x}}}$	Wald χ^2^	*P* value	OR	95%CI
Intercept	8.271	5.182	2.548	0.110		
PPD exposed levels						
Lower = 0					1	
Middle = 1	0.230	0.893	0.066	0.797	1.259	(0.219, 7.240)
Higher = 2	2.240	0.869	6.640	0.010	9.394	(1.710, 51.622)
Working age	0.031	0.042	0.553	0.457	1.031	(0.951, 1.119)
Gender						
Female = 0					1	
Male = 1	− 2.170	0.735	8.721	0.003	0.114	(0.027, 0.482)
Hair dye history						
Yes = 0					1	
No = 1	− 2.399	0.737	10.598	0.001	0.091	(0.021, 0.385)
Ethnic group						
Han = 0					1	
Others = 1	− 1.648	0.874	3.555	0.059	0.192	(0.035, 1.067)
Educational condition						
Primary school and lower level = 1					1	
Junior high school level = 2	− 1.242	1.127	1.214	0.271	0.289	(0.032, 2.632)
Senior middle school level = 3	− 3.197	1.337	5.718	0.017	0.041	(0.003, 0.562)
College and higher level = 4	− 3.418	1.544	4.904	0.027	0.033	(0.002, 0.675)
SF-36						
RP	− 0.034	0.047	0.519	0.471	0.967	(0.883, 1.059)
BP	0.055	0.024	5.337	0.021	1.056	(1.008, 1.106)
GH	− 0.052	0.025	4.206	0.040	0.950	(0.904, 0.998)
VT	− 0.007	0.031	0.051	0.821	0.993	(0.935, 1.054)
MH	− 0.008	0.042	0.038	0.845	0.992	(0.914, 1.076)
HT	− 0.017	0.015	1.389	0.239	0.983	(0.955, 1.012)

### Structural equation model for SF-36, VAS level and PPD exposed concentrations

A model with PPD exposed level predicting VAR level through SF-36 scores evidenced reasonable fit across indices (Supplemental Table 2). All indicators meet the reasonable reference value of the model (χ^2^/df = 2.215, GFI = 0.901, AGFI = 0.927, RMSEA = 0.092, IFI = 0.972, CFI = 0.969, PGFI = 0.593). The model with standardized path coefficients is presented in Supplemental Fig. 1. Direct paths were significant, PPD exposed level have positive effects on VAS level (β = 0.213, *p* < 0.001). Then PPD exposed level partly lead to side effects on total score of SF-36 (β = − 0.465, *p* = 0.002); on negative affect on VAR level (β = − 0.110, *p* < 0.001). The indirect path of hair dye history has negative affect on VAS level (β = − 0.221, *p* = 0.002), and PPD exposed levels via white blood cell count (β = − 0.952, *p* < 0.001), basophils (β = 0.015, *p* < 0.001), eosinophil (β = 0.110, *p* < 0.001) and hemoglobin (β = 3.349, *p* = 0.036) were all significant (*p* < 0.001), while neutrophil was non-significant (β = 0.080, *p* = 0.388). In the model, the indirect paths of PPD exposed level via pulmonary function indices showed FEV1% (β = − 5.051, *p* < 0.001) and FEV1/FVC% (β = − 6.333, *p* < 0.001) were significant, while FVC was non-significant (β = 1.188, *p* = 0.418).

## Discussion

In our study FEV1% was lower in higher exposure group compared with lower exposure group. The FEV1/FVC% was also lower in comparison to the higher exposure and middle exposure groups. PPD levels were negatively correlated with vitality and mental health. The structural equation model showed the positive effects of PPD on VAS level, and indicated partly negtive effects of PPD on total score of SF-36, respectively. Occupational PPD exposure might be associated with pulmonary injury, poor HRQL, and subjective pruritus of the workers.

Frequent contact with hair dyes production and working in the atmosphere with PPD aerosol can caused widespread health issues such as contact allergy^26^. In the present study, the blood biochemical indices, pulmonary function, HRQL and VAS of pruritus were compared among the workers who exposed to low dose PPD aerosol, because the results of air monitoring indicated that the mean concentration of PPD in different workshops were lower than the threshold-limited value-time-weighted average (0.1 mg/m^3^). The workers exposed in higher PPD concentration showed a lower white blood cell count and higher basophils, hemoglobin and eosinophils counts. An elevation of basophils, hemoglobin and eosinophils counts was similar to those found in neutrophilic dermatitis, apart from the pronounced eosinophilia. Numerous studies have reported that is a marker of hypersensitivity^27–29^. Moreover, the results of the present work agreed with most findings of the Sieben’s study that both PPD and its auto-oxidation product may stimulate lymphocyte proliferation, which is an important marker of inflammatory responses as well as allergic reactions and confirms the allergenicity of PPD^30,31^.

Our study was the first to explore whether PPD exposure induced the changes of pulmonary function examination. However, the sample size of our study was relatively too small, and the conclusion remains to be further verified. Both FEV1% and FEV1/FVC% were lower in the higher PPD exposure group, and FVC% was lower in the middle PPD exposure group than other two groups which are all more than 70% and are at normal levels^32^. In addition, the mean of all groups’ indices were in the normal range. The reason may cause this situation was that the hazard of low PPD dose with long-term exposure was relatively weaker. Some case report researches have also indicated that PPD poisoning could lead to respiratory distress^33,34^. However, these studies have not reported the effects of low PPD concentration on pulmonary function in the workplace. This finding implied that there might be an increased risk of respiratory disorders for workers in response to long-term PPD exposure.

Any health problems can cause a disruption in the normal life and have a substantial impact on the quality of life^35^, it seems important to assess the non-organ-specific manifestations such as HRQL. In the present study, we found that not only body pain, mental health and physical role were affected, but also the general health, vitality and reported health transition were affected for all workers. It has been suggested that these effects can have a long term effect of the acquisition of personality and health behaviors. Our results were similar to the conclusions reached by Mohammadi et al., who found that the workers exposed to higher levels of crystalline silica had lower values of pulmonary function indices and lower health-related quality of life^36^. Various studies have showed that age is a risk factor to physical health status^37,38^, because elders were usually affected by one or more chronic diseases and conditions^39^. However, the present study showed that the general health status, vitality and health transition were better in longer work-age workers. It might be explained by the healthy worker effect phenomenon that older workers usually have better health conditions than the general population^40,41^. People exposed in higher PPD levels have lower score in the dimensions of vitality and mental health. One reason for the results might because they engaged in different types of work that caused different PPD exposed concentration. The other reason for this could be the different ways to select subjects, with studies in this field based on retrospective analysis. The current study found that subjective feelings of bodily pain, mental health, and general health are not the same for men and women. As reported by previous study, general health and mental health were significantly associated with gender^10^.

In the light of the itching of skin seen as a subjective discomfort, the VAS of pruritus was used in this survey. The results of VAS levels in this survey are related to PPD exposed levels. A similar tendency was found that PPD is responsible for the majority of the allergic reactions to permanent hair color, among which the most common kind of allergic reactions is itchy^42^. It found that women were more likely to feel itching of skin than men. This is partly due to the physiological sensitivity of different gender to PPD after control the influence of gender^43^. Because VAS level is also affected by subjective perception, we analyze the impact of health-related quality of life and education condition on VAS level. It found that the higher the level of education, the lower the degree of itching. This may because that a high level of education will have a more comprehensive understanding of the dangers of PPD. The reciprocal and intricate relationship between the psyche and itch has been widely studied^44^. The augmentation of perception of itch owing to the interplay of psychogenic and emotional factors has been well studied^45^. Although pruritus itself is not recognized as a separate entity as such, DSM-V (Diagnostic and Statistical Manual of Mental Disorders, 5th edition by American Psychiatric Association) has already included a category of physical symptoms and related disorder. Pruritic conditions exacerbate the symptoms of itching. Itching and pain have the same receptors and have much in common^46^. Therefore, there is a certain relationship between the degree of body pain and VAS level.

We found direct paths from PPD exposure level to VAS level outcomes through one variable: influence of SF-36. Specifically, have hair dye history predicted negative affect on VAS level. PPD exposed may cause FEV1% and FEV1/FVC% decreased, and some influence to blood biochemical index such as white blood cell count, eosinophils, basophils and hemoglobin according to the results of structural equation model.

A key strength of our study is that we first studied the workers with the occupational exposure of low dose PPD as the research object, and measured the levels of occupational exposure to PPD in groups. Therefore, the exposure grouping in this study is more accurate and reasonable. An additional strength of our study is we comprehensively consider the physical and mental health of workers. This study analyzed the results of pulmonary function and HRQL according to the level of exposure. The results of our study also provided some useful data to support the formulation of occupational exposure limits (OELs) for PPD in workplace air, which has been included and issued as “Occupational exposure limits for hazardous agents in the workplace. Part 1: Chemical hazardous agents GBZ2.1-2019”. Further effective guidelines and measures will certainly be provided for the health protect of occupational population in China^47^.

There are some limitations in this study. First, the study is a cross-sectional study. Although a possible causal association has been established through structural equation modeling, further cohort studies and experimental studies are needed to verify these association. Secondly, the study was limited by its small sample size. Thus, large-scale studies are necessary in PPD occupationally exposed workers to gain a better understanding of the physiological and mental state of the workers. Moreover, there were different types of confounding factors for SF-36 scores and VAS levels: the socio-economic status, income, work type, and stress for life, therefore, further comprehensive studies should consider these factors.

## Conclusion

Our current study showed that occupational PPD exposure has an effect on subjective itching and health-related quality of life, and health-related quality of life mediates the effects of PPD exposure on subjective pruritus.

## Supplementary Information

Supplementary Tables.

Supplementary Figure 1.

## Data Availability

The data that support the findings of this study are available from the corresponding author upon reasonable request.
