# Supplementary material for: Association of p-phenylenediamine exposure with alterations of pulmonary function, pruritus and health-related quality of life in hair dye factory workers: a cross-sectional study
Source: Sci Rep. 2023 Feb 14;13:2623. doi: 10.1038/s41598-023-29721-7 (PMC9929233; doi:10.1038/s41598-023-29721-7)
Supplement: Supplementary file 1 — Supplementary Tables. [file 41598_2023_29721_MOESM1_ESM.docx]

**Supplemental Materials**

Association of p-phenylenediamine exposure with alterations of pulmonary function, pruritus and health-related quality of life in hair dye factory workers: a cross-sectional study

The supplemental material file includes:

Supplemental Table 1

Supplemental Table 2

Supplemental Figure 1

**Supplemental Table 1** PPD concentration in different types of work

| Workshop | PPD concentration in the air (mg/m^3^) | Individual PPD exposure concentration (mg/m^3^) |
| --- | --- | --- |
| Raw material configuration workshop | 0.01～0.19 | 0.025～0.047 |
| Workshop about add chemical | 0.0038～0.12 | 0.00033～0.015 |
| Workshop add PPD into the jar | 0.00089～0.04 | 0.00033～0.030 |
| Packaging workshop | ＜0.00089 | < 0.00033 |

**Supplemental Table 2** Summary of model fit

| Adaptability index | χ^2^/df | GFI | AGFI | RMSEA | IFI | CFI | PGFI | CAIC |
| --- | --- | --- | --- | --- | --- | --- | --- | --- |
| Reference value | 1-3 | >0.90 | >0.90 | <0.10 | >0.90 | >0.90 | >0.50 | CAIC_default_<CAIC_independence_  and CAIC_default_<CAIC_saturated_ |
| Model test value | 2.29 | 0.901 | 0.934 | 0.099 | 0.979 | 0.907 | 0.593 | 488.056<1432.971  and 890.503<890.503 |

**Supplemental Figure 1 legend**

**Path analysis of the effects of PPD exposure on SF-36 and VAS**

PPD exposure levels, hair dye history, blood routine index, pulmonary function index, SF-36 scores and VAS level, which were analyzed as the structural equation model by AMOSE software. The path coefficients in the figure are all standard path coefficients. e, errors; ↔There may have a certain common change effect; →Arrow points from reason to result. **p<*0.05; ***p<*0.001. Abbreviations: BP (bodily pain); GH (general health); RP (role-physical); MH (mental health); VT (vitality); HT (health transition); VAS (Visual analogue scale); SF-36 (36-item Short Form Health Survey); FEV1% (percentage of forced expiratory volume in one second); FVC% (percentage of forced vital capacity); FEV1/FVC % (ratio percentage of forced expiratory volume in one second /forced vital capacity).
