# Supplementary figures and images for: Association of p-phenylenediamine exposure with alterations of pulmonary function, pruritus and health-related quality of life in hair dye factory workers: a cross-sectional study
Source: Sci Rep. 2023 Feb 14;13:2623. doi: 10.1038/s41598-023-29721-7 (PMC9929233; doi:10.1038/s41598-023-29721-7)

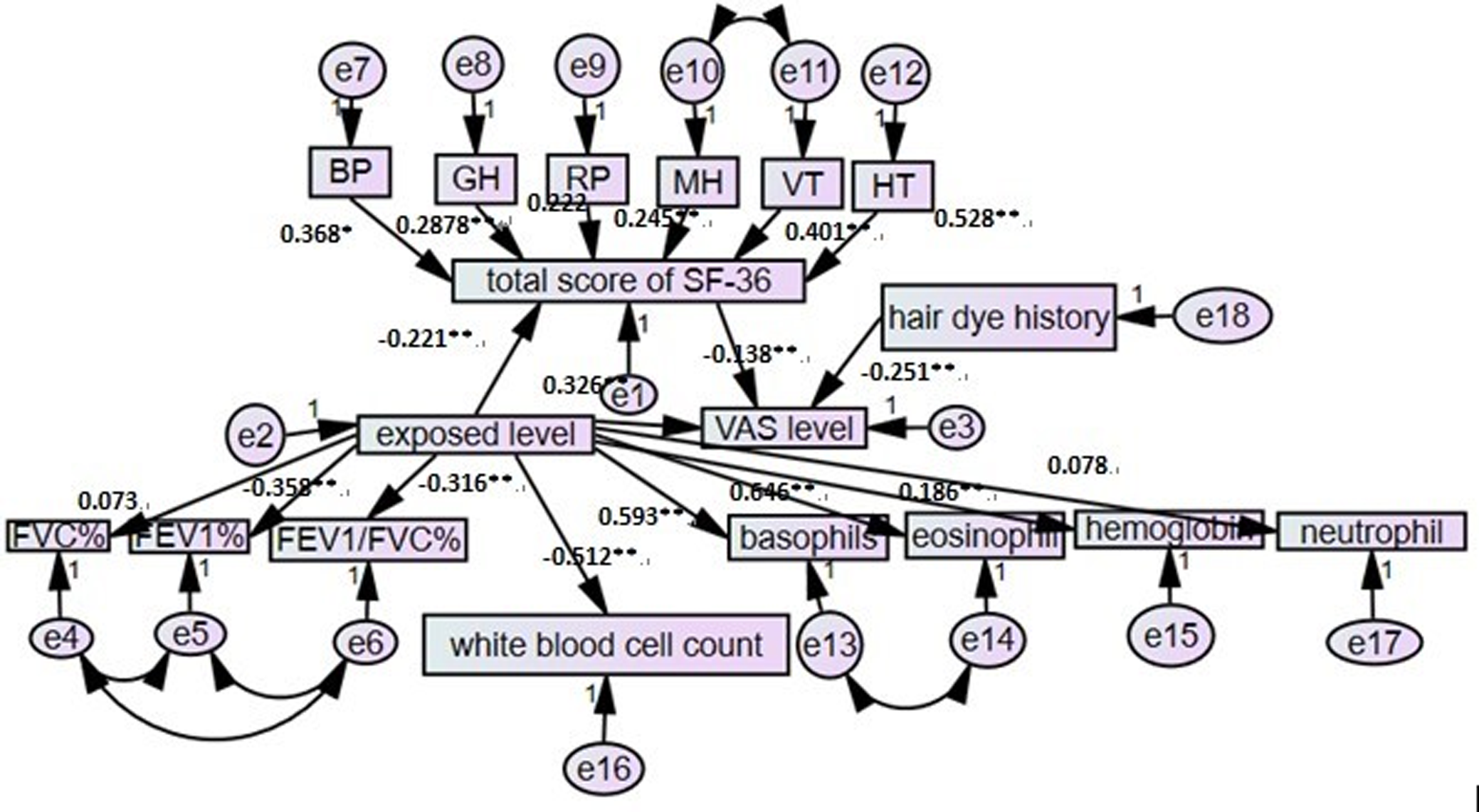

Supplement: Supplementary file 2 — Supplementary Figure 1. [file 41598_2023_29721_MOESM2_ESM.tif]
